# Childhood obesity and risk of Alzheimer’s disease: a Mendelian randomization study

**DOI:** 10.1186/s13690-024-01271-y

**Published:** 2024-03-18

**Authors:** Wenxiang Qing, Yujie Qian

**Affiliations:** 1 Department of Anesthesiology, Third Xiangya Hospital, Central South University, Changsha, 410013 China; 2 Department of Pediatrics, Xiangya Hospital, Central South University, Changsha, 410008 China

**Keywords:** Childhood obesity, Alzheimer's disease, Dementia, Mendelian randomization

## Abstract

**Background:**

Midlife obesity is a modifiable risk factor for Alzheimer’s disease. However, the association between childhood obesity and Alzheimer’s disease remains largely unknown. Therefore, we conducted a mendelian randomization analysis (MR) to assess the causal link between childhood obesity and Alzheimer’s disease.

**Methods:**

Using summary statistics from publicly available genome-wide association studies (GWAS) database, we explored the genetic link between childhood obesity and Alzheimer’s disease through a two-sample MR. The primary analysis employed the inverse-variance weighted (IVW) method. To complement our findings, we also employed MR-Egger, weighted median, simple model, and weighted model methods for MR estimates. Furthermore, we conducted Cochrane’s Q-statistic test, Egger intercept test, and a leave-one-out sensitivity test to ensure the robustness and reliability of our results.

**Results:**

The IVW analysis yielded non-significant results, indicating no significant genetic association between childhood obesity and Alzheimer’s disease (OR = 0.958, 95% CI = 0.910–1.008, *p* = 0.095). Consistent with this, the results from MR-Egger, the weighted median, simple model, and weighted model approaches all supported these findings. Furthermore, we did not detect any signs of heterogeneity or pleiotropy, and our leave-one-out analysis confirmed that no single nucleotide polymorphisms had a substantial impact on the reliability of our results.

**Conclusions:**

The evidence from our MR analyses suggests that there is no causal effect of childhood obesity on the risk of Alzheimer’s disease.

**Supplementary Information:**

The online version contains supplementary material available at 10.1186/s13690-024-01271-y.

**Table:** 

**Text box 1. Contributions to the literature**	
• Public health policies targeting Alzheimer’s disease risk factors among early life course-related traits are urgently needed.	
• Healthcare systems should give due importance to the impact of childhood obesity on adulthood and later life.	
• There is limited evidence on contributions of healthcare systems and public health policies in childhood obesity in Alzheimer’s disease.	

## Background

Alzheimer’s disease is a progressive neurodegenerative disorder that manifests as a gradual decline in cognitive function, ultimately resulting in severe impairment and loss of independence. This condition affects more than 27 million individuals and stands as the predominant form of dementia among the elderly population [1, 2]. Therefore, Alzheimer’s disease has emerged as a significant public health concern and a top priority for research. Despite ongoing efforts, the precise cause of Alzheimer’s disease remains elusive. Notably, the primary risk factors for Alzheimer’s disease include age, genetics, and family history, all of which are non-modifiable [3, 4]. Growing evidence supports twelve modifiable risk factors for dementias modelled by the 2020 *Lancet* Commission [5]. It has been estimated that approximately one-third of all global cases of Alzheimer’s disease can be attributed to modifiable risk factors [6]. Moreover, a reduction in these modifiable risk factors by 10–25% could potentially prevent up to 3 million cases of Alzheimer’s disease globally [7]. These emphasize the potential impact of addressing these modifiable factors in reducing the overall burden of the disease. One of the identified twelve modifiable risk factors for dementia is midlife (45–65 years) obesity, which has been found to be predictive of future Alzheimer’s disease [5, 8–11]. Research indicates that Alzheimer’s disease may start developing 20 years or more before symptoms arise [12]. In the 2017 *Lancet* Commission, a life-course model was proposed to explore potentially modifiable risks for dementia [13]. Considering the significance of the life course in understanding risks, it is important to investigate whether childhood obesity can predict the development of Alzheimer’s disease in the future.

Most of the recent data has primarily examined the link between obesity and Alzheimer’s disease in adults. Numerous potential mechanisms have been proposed to explain the connection between obesity and Alzheimer’s disease, including systemic inflammation, activation of astroglia, plaque deposition, and impaired plaque clearance [14]. With the increased prevalence of obesity and the earlier onset of obesity in the population, there may be a significant rise in the incidence and prevalence of Alzheimer’s disease among younger individuals [15]. Childhood obesity, which is highly prevalent, has been linked to various health issues, including cognitive impairment during childhood [16, 17]. Longitudinal studies examining the association between childhood obesity and cognitive function in midlife have revealed that childhood obesity has a detrimental effect on cognition later in life [18]. These findings suggest that factors influencing cognitive function in midlife may already be present during childhood. Additionally, research supports a connection between lower midlife cognitive function and the development of dementia in older age [19]. Based on these observations, it is plausible to speculate that childhood obesity increases the risk of cognitive impairment in midlife and ultimately contributes to the development of Alzheimer’s disease.

Clarifying the causal link between childhood obesity and Alzheimer’s disease is of utmost importance, as it will enable us to better prepare for the substantial influx of future Alzheimer’s disease patients.

## Methods

### Study design and data sources

**Fig. 1:**
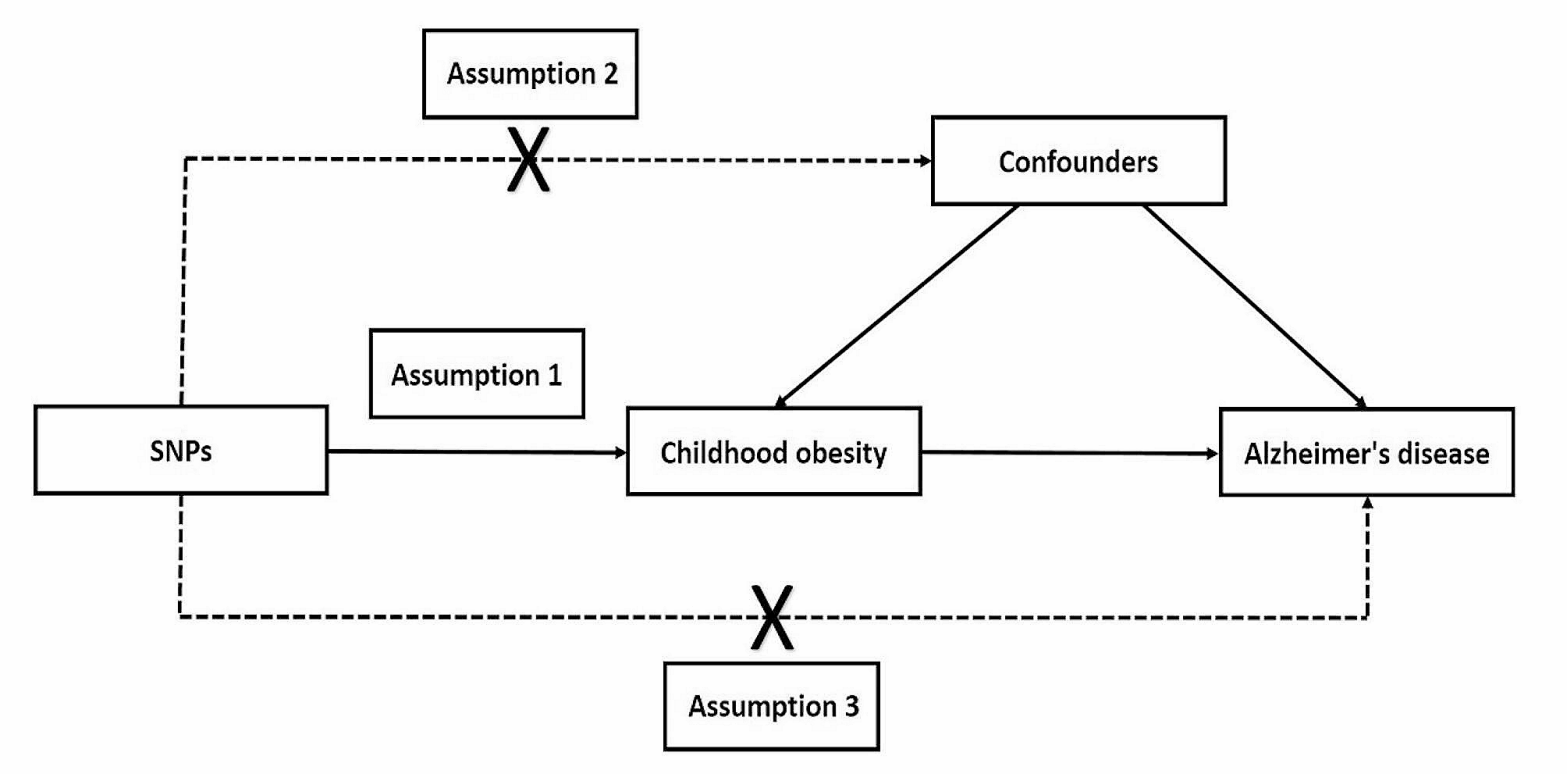
Schematic view of design and three key assumptions of the Mendelian randomization study

We conducted a two-sample MR study using publicly available data from genome-wide association studies (GWAS). Since we relied on existing data, no additional ethical approvals were required. In MR studies, genetic information is utilized as instrumental variables (IVs) [20]. In our study, childhood obesity was considered as the exposure, Alzheimer’s disease as the outcome, and single nucleotide polymorphisms (SNPs) as the IVs. To obtain SNPs associated with childhood obesity, we utilized data from the Early Growth Genetics (EGG) consortium. This dataset included 5,530 patients (in the 95th percentile of body mass index (BMI) for their age [21]) and 8,318 controls (in the below 50th percentile of BMI) from European children [22]. Exposure to SNPs of childhood BMI was obtained from another publicly available GWAS dataset with a total sample size of 61,111 European children aged between 2 and 10 years [23]. The data for the outcome, Alzheimer’s disease, was sourced from another publicly available GWAS dataset, comprising 21,982 patients and 41,944 controls from European populations [24]. To achieve a more stable result, we conducted the primary MR analyses in other datasets of Alzheimer’s disease. Dataset for Alzheimer’s disease-repeated comprised 39,106 patients and 46,828 controls from European populations [25]. A schematic view of the study design is shown in Fig. 1. The rationale of our MR design was based on three assumptions: (1) genetic variation is robustly associated with childhood obesity; (2) genetic variation is not associated with other confounders; (3) genetic variation only influences the outcome by childhood obesity.

### Genetic instrumental variables selection

To ensure effective instrumental variables (IVs) that fulfill the three key MR assumptions, we performed a set of quality control techniques. Firstly, we identified 6 SNPs as IVs representing childhood obesity to conduct MR analysis in the threshold of *p* < 5 × 10^− 8^. As MR studies require at least 10 IVs [26, 27], we extract 15 SNPs using a threshold of *p* < 5 × 10^− 6^ [28, 29]. To eliminate weak instrument bias, F statistics were calculated and an F < 10 was considered dubious bias [28]. To validate these SNPs, we conducted phenome-wide association studies (pheWAS) utilizing catalog databases, confirming that there were no potential relationships between these SNPs and any measured or unmeasured confounding factors [30, 31]. Secondly, we applied a minor allele frequency (MAF) threshold of 0.01 to ensure that the selected SNPs were meaningful in terms of their frequency within the population. Thirdly, we utilized a clumping process with an r2 < 0.001 and a clumping distance of 10,000 kb to select SNPs with low linkage disequilibrium (LD) levels. Lastly, we ensured that the effects of the selected SNPs on the exposure (childhood obesity) corresponded to the same allele as the effect on the outcome (Alzheimer’s disease). Any palindromic SNPs were excluded from our IVs to maintain the integrity of our analysis.

### Two-sample MR analysis

In our study, the main MR analysis was conducted using the classic inverse-variance weighted (IVW) method to evaluate the causal effect of childhood obesity on Alzheimer’s disease. The IVW method is known for providing a stable and accurate causal evaluation when there is no evidence of directional pleiotropy [20, 32]. In addition to the IVW method, we also employed other MR analysis methods to estimate causal effects. These methods included MR-Egger regression, weighted median, simple model, and weighted model methods. The MR-Egger regression method is particularly useful when there is potential for directional pleiotropy. This method allows for the assessment of causal effects even in the presence of unbalanced horizontal pleiotropy [33]. The weighted median method estimates the causal effect by taking the median of the individual instrumental variable estimates, allowing for robustness against potential violations of the instrumental variable assumptions [34]. Similarly, the simple model [34] and weighted model [35] methods provide alternative approaches to estimate causal effects, offering additional insights in cases where assumptions underlying the IVW method may be violated.

By employing multiple MR analysis methods, we aimed to obtain a comprehensive understanding of the causal relationship between childhood obesity and Alzheimer’s disease, considering the strengths and limitations of each approach.

### Sensitivity analyses

To assess the heterogeneity and potential pleiotropy among the IVs, we conducted Cochrane’s Q-statistic test and Egger intercept test. These tests allow us to examine the presence of heterogeneity in causal estimates and detect potential bias caused by horizontal pleiotropy. Additionally, we performed leave-one-out analyses to examine the robustness of the MR results. This analysis involves systematically excluding one IV at a time and re-estimating the causal effect to assess the influence of individual IVs on the overall estimate. In all sensitivity analyses, we utilized a *p*-value threshold of 0.05 to determine statistical significance.

All MR and sensitivity analyses were conducted in R (version 4.3.1) using the Two-Sample MR package (version 0.5.7).

## Results

### SNPs used as instrumental variables

We identified independent SNPs from the GWAS dataset following the methodology described in the previous section. The remaining 15 SNPs were included to establish the genetic IVs for childhood obesity (Table 1). F statistics were calculated to estimate weak instrument bias considering the relatively relaxed threshold in childhood obesity, and an F < 10 was considered dubious bias.

**Table 1:** Childhood obesity SNPs used to construct the instrument variables

chr	Position	SNP	Effect Allele	Other Allele	Beta	SE	*p* value	F
1	74,977,870	rs1040070	C	G	-0.1492	0.0269	2.77E-08	30.75887
1	177,913,519	rs10913469	C	T	0.1773	0.033	7.99E-08	28.86203
2	638,144	rs4854344	T	G	0.2445	0.0351	3.22E-12	48.51553
2	25,150,116	rs6752378	A	C	0.1695	0.0262	1.05E-10	41.84794
4	45,175,691	rs13130484	T	C	0.1434	0.0272	1.30E-07	27.79059
4	113,311,790	rs4833407	A	C	0.1226	0.0265	3.88E-06	21.40063
4	130,731,284	rs4864201	C	T	-0.1355	0.0281	1.41E-06	23.24894
5	66,149,113	rs28636	T	C	-0.1474	0.0316	3.07E-06	21.75495
12	50,247,468	rs7138803	A	G	0.1672	0.0271	6.50E-10	38.06021
13	54,064,981	rs9568856	A	G	0.1909	0.0395	1.36E-06	23.35366
16	53,825,488	rs9941349	T	C	0.1978	0.0267	1.16E-13	54.87409
17	46,669,430	rs9299	T	C	0.1343	0.0282	1.91E-06	22.67729
18	57,839,769	rs571312	A	C	0.1986	0.0309	1.25E-10	41.30273
18	38,765,659	rs17697518	T	C	0.1855	0.0389	1.85E-06	22.73662
19	34,315,896	rs256335	T	C	0.1214	0.0262	3.72E-06	21.46703

Chr, chromosome; SNP, single-nucleotide polymorphism; Beta, beta coefficient; SE, standard error

### MR analysis of childhood obesity and Alzheimer’s disease

The MR analysis conducted in this study aimed to investigate the causal relationship between childhood obesity and Alzheimer’s disease risk. First, we analyzed the causal relationship between childhood BMI and Alzheimer’s disease risk. There is no causal relationship between childhood BMI and the risk of Alzheimer’s disease using the IVW analysis (Odds Ratio [OR] 0.989, 95% Confidence Interval [CI] [0.846, 1.152], *p* = 0.886). This finding was consistent across different MR analysis methods, including MR-Egger (OR 0.791, 95% CI [0.419, 1.494], *p* = 0.482), simple model (OR 0.905, 95% CI [0.684, 1.196], *p* = 0.492), weighted model (OR 0.892, 95% CI [0.701, 1.135], *p* = 0.368), and weighted median (OR 0.916, 95% CI [0.775, 1.084], *p* = 0.308) analyses (Table S1, Figure S1).

**Fig. 2:**
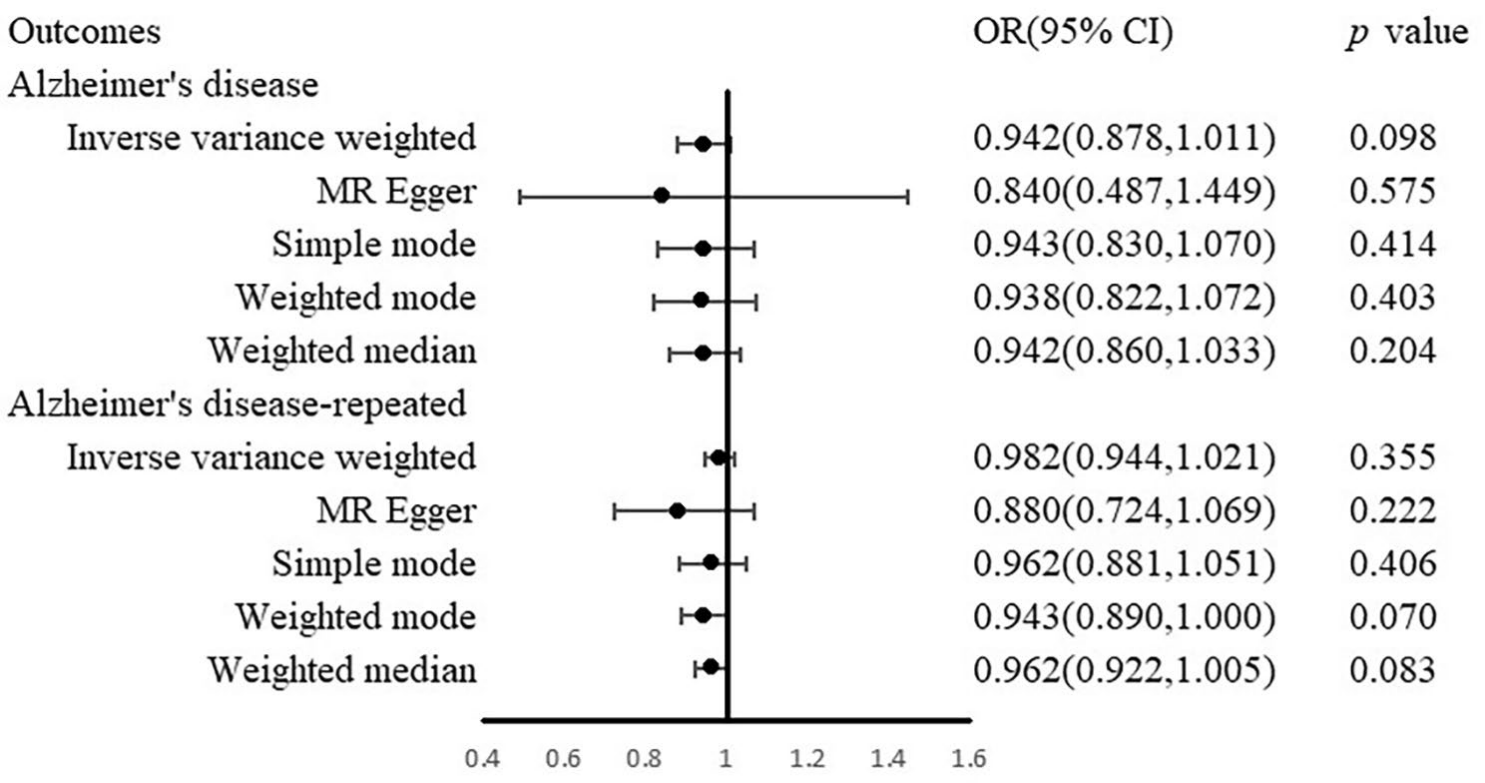
Associations between genetically predicted childhood obesity and Alzheimer’s disease risk. OR, odds ratio; CI, confidence interval

**Fig. 3:**
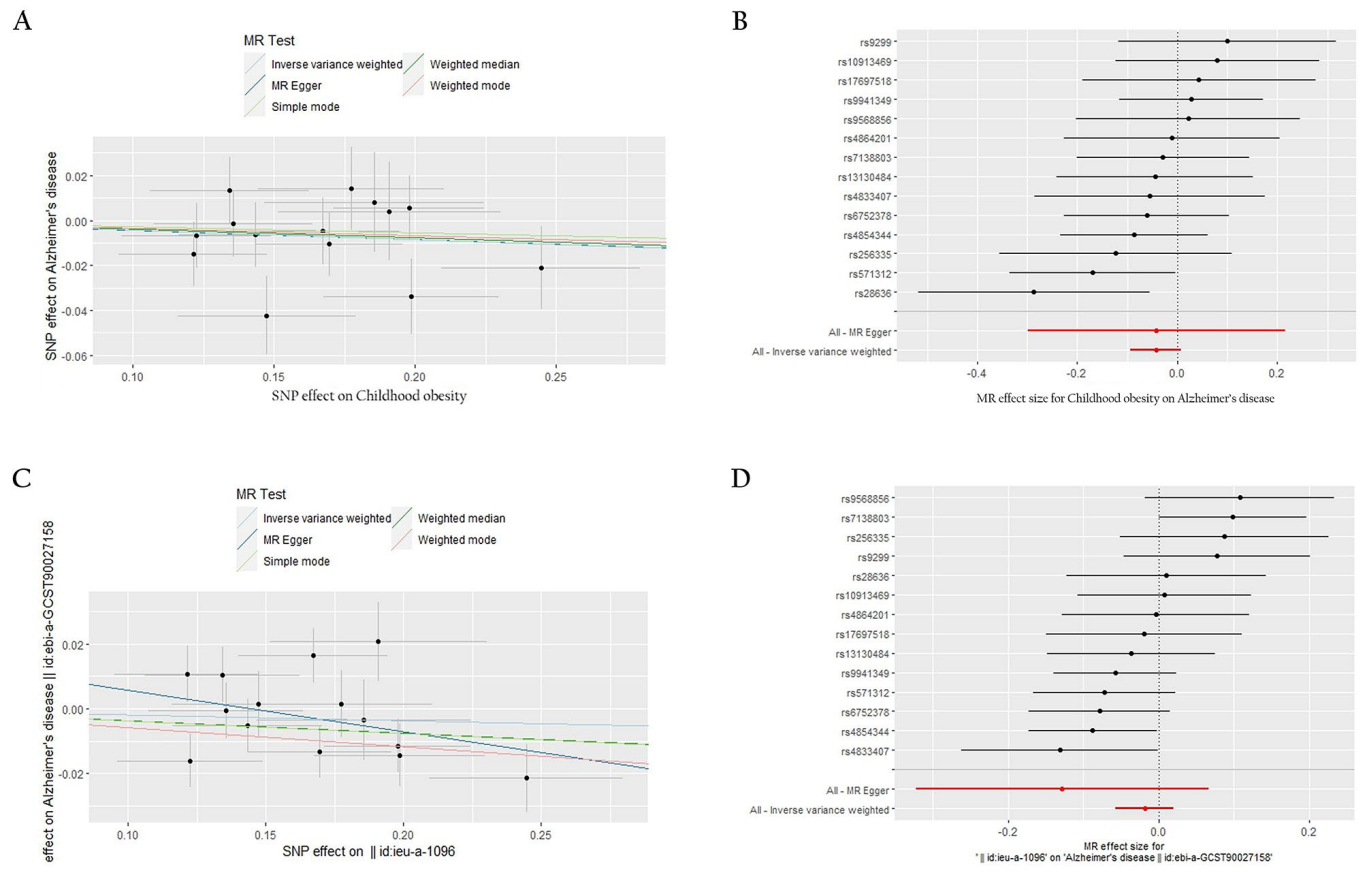
Plots of MR estimates of the causal relationship between childhood obesity and Alzheimer’s disease. **(A)** The scattered plot of SNPs associated with childhood obesity and their risk on Alzheimer’s disease. **(B)** Forest plot of SNPs associated with childhood obesity and their risk on Alzheimer’s disease. **(C)** The scattered plot of SNPs associated with childhood obesity and their risk on Alzheimer’s disease in other datasets. **(D)** Forest plot of SNPs associated with childhood obesity and their risk on Alzheimer’s disease in other datasets. SNPs, single nucleotide polymorphisms

However, we remain open to the possibility of a non-linear causal relationship between childhood BMI and Alzheimer’s disease. Then we conducted childhood obesity as instrumental variables and no significant causal effect of the genetic risk variants on Alzheimer’s disease was observed using the IVW analysis (Odds Ratio [OR] 0.958, 95% Confidence Interval [CI] [0.910, 1.008], *p* = 0.095). This finding was consistent across different MR analysis methods, including MR-Egger (OR 0.959, 95% CI [0.741, 1.241], *p* = 0.755), simple model (OR 0.973, 95% CI [0.866, 1.094], *p* = 0.658), weighted model (OR 0.967, 95% CI [0.865, 1.081], *p* = 0.563), and weighted median (OR 0.962, 95% CI [0.898, 1.030], *p* = 0.265) analyses (Figs. 2 and 3). To achieve a more stable result, we repeated the primary MR analyses in other datasets of Alzheimer’s disease and got the same results (Figs. 2 and 3). These results suggest that there is no causal relationship between childhood obesity and the risk of Alzheimer’s disease.

### Sensitivity analysis

**Fig. 4:**
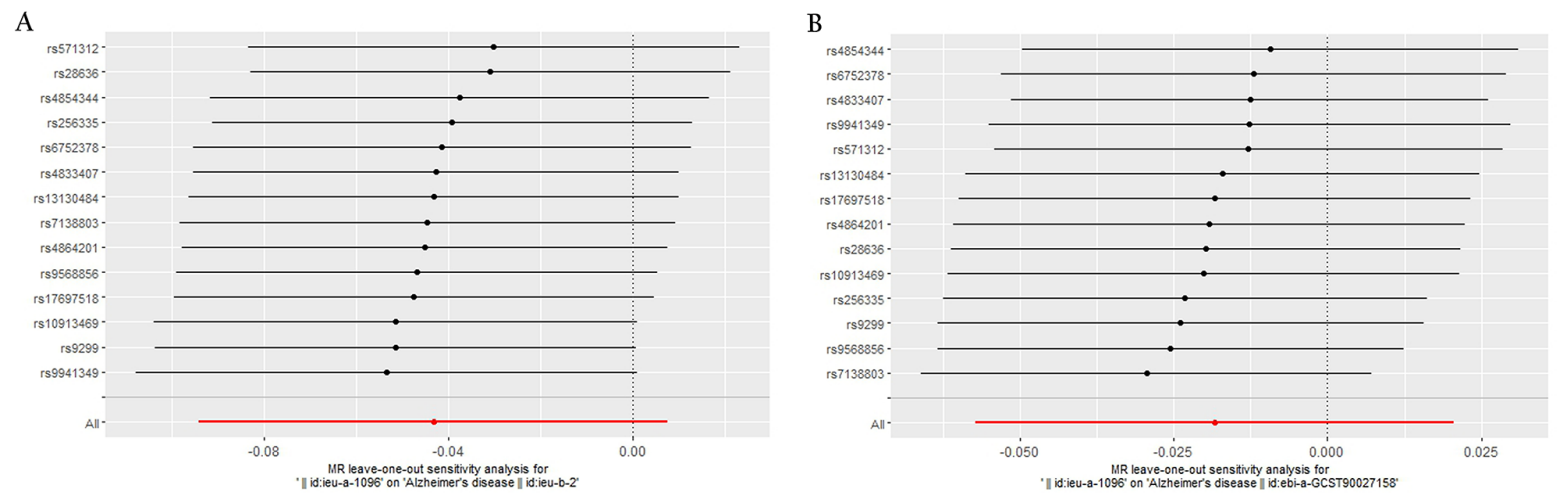
A leave one-out pleiotropy test was performed for each SNP to determine the robustness of the MR test. **(A)** Childhood obesity - Alzheimer’s disease. **(B)** Childhood obesity - Alzheimer’s disease repeated. SNP, single-nucleotide polymorphism

To ensure the reliability of our findings, we conducted a sensitivity analysis. The IVW and MR-Egger tests for heterogeneity revealed no statistically significant heterogeneity in the MR analysis results between childhood obesity and Alzheimer’s disease (*p* > 0.05), as shown in Table 2. This indicates that our results are not confounded by other factors within the populations grouped by instrumental variables. Additionally, we investigated the presence of horizontal pleiotropy in our MR analysis. The results indicated that there was no significant horizontal pleiotropy effect (*p* > 0.05), as presented in Table 2. Figure 4 presents the outcomes of this leave-one-out sensitivity analysis. It visually represents the effects of excluding each SNP on the estimated causal relationship between childhood obesity and Alzheimer’s disease. By observing the changes in the results with the removal of each SNP, we can assess the robustness and reliability of the overall findings.

**Table 2:** Heterogeneity and horizontal pleiotropy tests for causal effects of childhood obesity on Alzheimer’s disease

Method	Heterogeneity			Horizontal pleiotropy		
	Q	df	*p* value	Egger intercept	Egger SE	*p* value
Alzheimer’s disease						
MR Egger	12.29791	12	0.422	0.000	0.022	0.992
IVW	12.29801	13	0.503			
Alzheimer’s disease-repeated						
MR Egger	21.23890	12	0.077	0.019	0.016	0.282
IVW	23.48531	13	0.087			

BMI, body mass index; Q, Cochrane’s Q; IVW, inverse variance weighted

## Discussion

Based on our MR study findings, we do not find support for a causal association between genetically predicted childhood obesity and Alzheimer’s disease risk.

It is worth noting that obesity and Alzheimer’s disease exhibit similarities in terms of shared features such as chronic inflammation, increased oxidative stress, and impaired energy metabolism [36]. However, the results of previous observational studies examining the association between obesity and Alzheimer’s disease have shown inconsistencies, particularly in relation to age. One comprehensive review of 19 longitudinal studies, including a large cohort of 589,649 participants with up to 42 years of follow-up, reported an association between midlife obesity and an increased risk of dementia in late life [37]. Additionally, a prospective population-based cohort study found that midlife obesity was predictive of an earlier onset of Alzheimer’s disease and higher levels of neuropathology. Conversely, maintaining a healthy BMI during midlife appeared to delay the onset of Alzheimer’s disease [38]. However, when focusing on obesity in late life, the relationship with Alzheimer’s disease appears to have different dynamics. A population-based prospective cohort study reported a reversed risk estimation, indicating that higher baseline BMI in late life was associated with a reduced risk of Alzheimer’s disease [39]. Similarly, another prospective study found that obesity in late life was associated with a lower risk of dementia compared to individuals with a normal BMI [40].

Although midlife obesity has been found to be predictive of future Alzheimer’s disease, 70% of obese adults were not obese in childhood or adolescence [41]. Moreover, the majority of adult obesity-related morbidity occurs in adults who were not obese during childhood [42]. Currently, little is known about the potential risk of childhood obesity in relation to Alzheimer’s disease in later life, despite the fact that childhood obesity can lead to significant health consequences that often become apparent during adulthood [43]. One large cohort study demonstrated a negative effect of childhood obesity on midlife cognitive function [18]. We found no causal association of childhood BMI with Alzheimer’s disease, which consistent with two previous MR studies about causal relationship of early life course-related traits with psychiatric disorders [44, 45]. However, we remain open to the possibility of a non-linear causal relationship between childhood BMI and Alzheimer’s disease. The objective of our current research is to investigate the potential associations between genetically determined childhood obesity and Alzheimer’s disease. This perspective is important to consider in the broader context of understanding long-term health implications of childhood obesity and its potential influence on later-life cognitive health. Due to the strong correlation between childhood obesity and the development of diabetes and hypertension in adulthood [42], both of which are considered modifiable risk factors for Alzheimer’s disease [5]. It becomes challenging to ascertain the independent effects of childhood obesity. By using genetically predicted childhood obesity as instrumental variables, we aimed to minimize the potential influence of confounders. Consequently, our study found no causal association between genetically predicted childhood obesity and the risk of Alzheimer’s disease.

## Conclusions

In contrast to midlife obesity, which has been identified as a modifiable risk factor for Alzheimer’s disease, our findings suggest that childhood obesity does not have a causal association with the risk of developing Alzheimer’s disease. This discrepancy highlights the possibility that obesity during different stages of life may be differentially associated with the risk of Alzheimer’s disease. Future studies should consider examining the potential mechanisms underlying the differing associations between obesity during different life periods and Alzheimer’s disease risk. Such investigations can contribute to a more comprehensive understanding of the impact of obesity on the development and progression of Alzheimer’s disease.

### Limitations

However, there are limitations to this MR study. First, few SNPs fell under the standard bioinformatic threshold of *p* < 5 × 10^− 8^. Consequently, we opted to select SNPs using a less stringent significance level of 5 × 10^− 6^, as employed in previous studies [28, 29]. Therefore, weak instrumental bias may exist, we calculated F statistics to assess the risk of such bias and an F < 10 was considered dubious bias. Second, due to data limitations in the original studies, we were unable to conduct subgroup analyses based on demographic factors such as gender, ethnicity, and education. Third, it is important to highlight that the only publicly available GWAS dataset for childhood obesity did not provide specific information on weight, height, and abdominal circumference. Consequently, we were unable to conduct subgroup analyses based on obesity classifications. This limitation restricted our ability to investigate potential variations in the relationship between different obesity classes and Alzheimer’s disease.

### Electronic supplementary material

Below is the link to the electronic supplementary material.

Supplementary Material 1

Supplementary Material 2

## Data Availability

No datasets were generated or analysed during the current study.

## Abbreviations

MR: Mendelian randomization
GWAS: Genome-wide association studies
IVW: Inverse-variance weighted
IVs: Instrumental variables
SNPs: Single nucleotide polymorphisms
BMI: Body mass index
EGG: Early Growth Genetics
