# Supplementary material for: Childhood obesity and risk of Alzheimer’s disease: a Mendelian randomization study
Source: Arch Public Health. 2024 Mar 18;82:39. doi: 10.1186/s13690-024-01271-y (PMC10949616; doi:10.1186/s13690-024-01271-y)
Supplement: Supplementary file 1 — Supplementary Material 1 [file 13690_2024_1271_MOESM1_ESM.docx]

Table 2. Childhood BMI SNPs used to construct the instrument variables.

| chr | Position | SNP | Effect Allele | Other Allele | Beta | SE | p value |
| --- | --- | --- | --- | --- | --- | --- | --- |
| 1 | 110082551 | rs41279738 | G | T | 0.1199 | 0.0211 | 1.30E-08 |
| 1 | 177889480 | rs543874 | G | A | 0.0793 | 0.0099 | 1.62E-15 |
| 1 | 72754314 | rs61765651 | T | C | -0.0584 | 0.0102 | 9.50E-09 |
| 1 | 74997762 | rs12042908 | G | A | -0.0586 | 0.0077 | 2.77E-14 |
| 2 | 207064335 | rs114670539 | T | C | 0.0991 | 0.0179 | 3.16E-08 |
| 2 | 621558 | rs939584 | T | C | 0.1066 | 0.0102 | 8.85E-26 |
| 2 | 25141538 | rs11676272 | G | A | 0.075 | 0.0079 | 2.37E-21 |
| 4 | 45179883 | rs12641981 | T | C | 0.044 | 0.008 | 4.19E-08 |
| 4 | 103188709 | rs13107325 | T | C | 0.0953 | 0.0173 | 3.51E-08 |
| 6 | 50791640 | rs2076308 | C | G | 0.0697 | 0.0099 | 1.58E-12 |
| 8 | 28061823 | rs62500888 | G | A | -0.0472 | 0.0076 | 6.91E-10 |
| 11 | 27723334 | rs56133711 | A | G | 0.0566 | 0.0089 | 2.00E-10 |
| 12 | 50247468 | rs7138803 | A | G | 0.0729 | 0.008 | 7.12E-20 |
| 13 | 54104968 | rs4477562 | T | C | 0.0802 | 0.0112 | 8.29E-13 |
| 16 | 53813367 | rs17817449 | G | T | 0.0683 | 0.008 | 1.69E-17 |
| 16 | 19980931 | rs7199285 | T | C | -0.0647 | 0.0101 | 1.34E-10 |
| 18 | 57839769 | rs571312 | A | C | 0.059 | 0.0093 | 2.00E-10 |
