# Supplementary figures and images for: Childhood obesity and risk of Alzheimer’s disease: a Mendelian randomization study
Source: Arch Public Health. 2024 Mar 18;82:39. doi: 10.1186/s13690-024-01271-y (PMC10949616; doi:10.1186/s13690-024-01271-y)

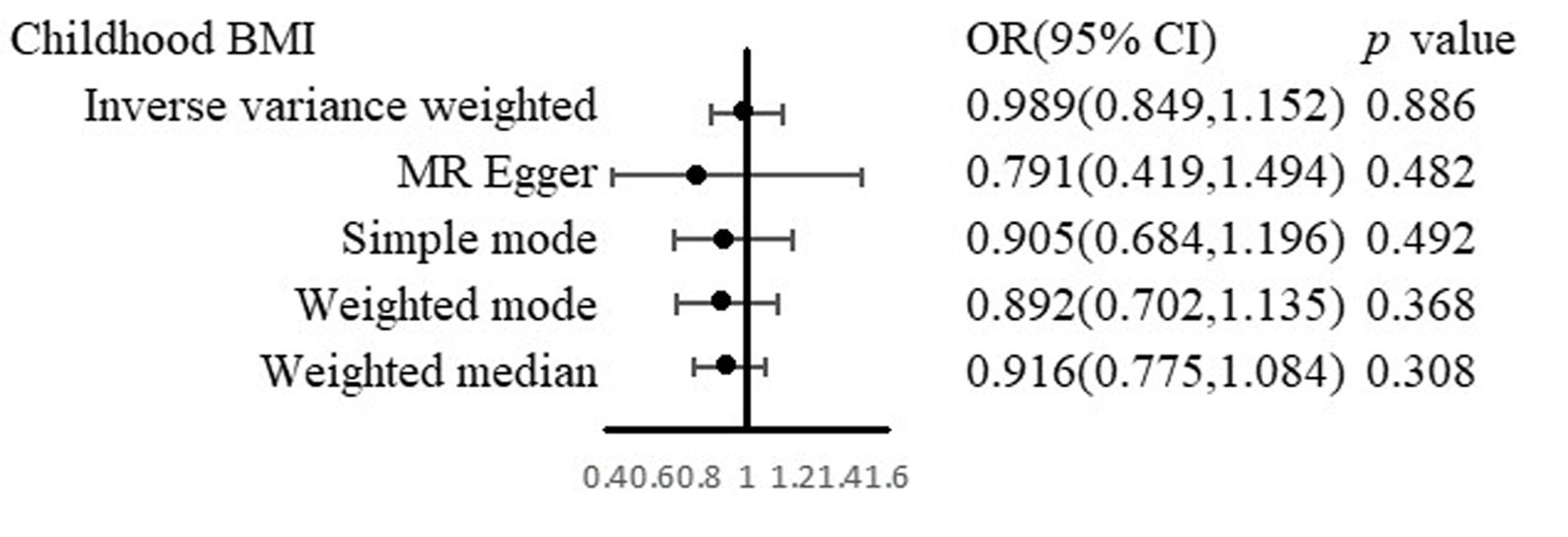

Supplement: Supplementary file 2 — Supplementary Material 2 [file 13690_2024_1271_MOESM2_ESM.jpg]
